# The Association between Alcohol Consumption Patterns and Health-Related Quality of Life in a Nationally Representative Sample of South Korean Adults

**DOI:** 10.1371/journal.pone.0119245

**Published:** 2015-03-18

**Authors:** KyungHee Kim, Ji-Su Kim

**Affiliations:** Red Cross College of Nursing, Chung-Ang University, Seoul, Republic of Korea; Örebro University, SWEDEN

## Abstract

This study examined the relationship between alcohol consumption and health-related quality of life (HRQOL) in a nationally representative sample of middle-aged to older South Koreans. Data collected from 3,408 men and 3,361 women aged ≥ 40 years were obtained from the 2010 and 2011 Korea National Health and Nutrition Examination Survey. Based on the World Health Organization guidelines, the participants were categorized into zones I (0–7), II (8–15), III (16–19), or IV (20–40) according to their Alcohol Use Disorders Identification Test (AUDIT) scores, with a higher zone indicating a higher level of alcohol consumption. Data collected from the AUDIT and EuroQol 5-Dimension (EQ-5D) test were subjected to multiple regression analysis in order to examine the relationship between alcohol consumption patterns and health-related quality of life, and to identify intersex and interzone differences. Significant intersex differences were found for the mean total AUDIT and EQ-5D scores and the proportion of participants rating their pain/discomfort and impairment in mobility and usual activities as “moderate” or “severe” (p < 0.001). The analysis of the EQ-5D scores by alcohol consumption pattern and sex suggested the existence of an inverted U-shaped relationship between the total AUDIT and EQ-5D scores. The HRQOL of moderate alcohol drinkers was higher than that of non-drinkers and heavy drinkers. The results of this study will be valuable in designing appropriate interventions to increase the HRQOL impaired by the harmful use of alcohol, in comparing HRQOL among different countries, and in implementing alcohol-related health projects.

## Introduction

The mean alcohol consumption per person in South Korea for 2011 was reportedly 8.9 L, which was relatively low compared to the mean of 9.5 L per person for all members of the Organization of Economic Cooperation and Development (OECD) [1]. However, as these figures include individuals who do not consume alcohol for religious and/or health-related reasons, the average alcohol intake among the general population is likely higher. In fact, the rate of alcohol consumption, as well as the negative consequences associated with its excessive consumption, has been increasing steadily in South Korea [2–4].

In general, the relationship between alcohol consumption and health outcomes is complex and multidimensional [5]. A moderate level of alcohol consumption may be positive in terms of stress relief and psychological health; however, excessive consumption of alcohol has been identified as a causal factor in a number of diseases, as well as a precursor to injury and violence [6,7]. Alcohol consumption has been reported to be influenced by sociological, environmental, and psychological factors. In particular, it has been strongly associated with health-related behaviors, such as smoking and exercise, along with health status [3]. Based on reports that the risk of harmful alcohol consumption may increase with the severity of negative health-related behaviors [8,9], the existence of a relationship between harmful alcohol consumption and poor health-related quality of life (HRQOL) has been hypothesized. The HRQOL is a self-report measure of physical and mental health reflecting physical, psychological, and social wellbeing [10,11]. It has been used widely in the design of health policies, and as an index of the physical and mental health of the general population [12, 13].

A number of previous studies have reported a relationship between alcohol consumption patterns and HRQOL in various groups, including alcoholics [14–16]. However, the initial results of studies on the relationship between alcohol consumption patterns and HRQOL from multiple countries have been inconsistent [5,17,18]. A major challenge in such research is the use of different methods of categorizing alcohol consumption patterns, which creates difficulty in comparing study results, and possibly produces differing results regarding the potential positive effects of moderate alcohol consumption on HRQOL [5]. Another major challenge specific to South Korea is that men and women in this country have distinctive differences in terms of alcohol consumption patterns and health status, including chronic disease prevalence and the use of medical resources, as well as their smoking, educational, marital, and financial status [19]. A particularly striking difference is that the rate of alcoholism is twice as high in men compared to women in South Korea [2, 20].

The consideration of such differences in a study of alcohol consumption in South Korea would require the use of a sex-differentiated approach using instruments designed to standardize internationally comparable data from a representative sample. The objective of this study was to employ such an approach to examine and compare the general characteristics, health-related behaviors, and health status of South Koreans aged ≥ 40 years with different patterns of alcohol consumption in order to analyze the relationship between their alcohol consumption and HRQOL.

## Materials and Methods

### Ethics Statement

This study’s protocol for performing a secondary analysis of the 2010–2011 KNHANES data was reviewed and approved by the Institutional Review Board (Approval No. 2010–02CON-21-C and 2011–02CON-06-C) of the Korea Centers for Disease Control and Prevention (KCDC). We requested permission from the KCDC to the use the KNHANES survey results for research purposes, and we submitted a data use plan and posted a written pledge on the KNHANES homepage [21]. Informed consent was obtained from all of the participants when the 2010 and 2011 KNHANES were conducted.

### Study Population

The data examined in this study were obtained during the 2010 and 2011 Korea National Health and Nutrition Examination Survey (KNHANES), which collected statistical data regarding national health status and health-related conditions that were reported to the World Health Organization (WHO) and OECD. The KNHANES consisted of a health interview survey, a health examination survey, and a nutrition survey. The data collected by the Alcohol Use Disorders Identification Test (AUDIT) and EuroQol 5-Dimension (EQ-5D) test, both of which are international standardized instruments, were subjected to multiple regression analysis to investigate the relationship between alcohol consumption patterns and HRQOL in a nationally representative sample of South Korean men and women aged ≥40 years.

The period V KNHANES (2010–2012) used a rolling survey sampling method to select participants who were representative of the civilian, non-institutionalized population of South Korea. To ensure that the sample was nationally representative, primary sampling units were selected in stage 1, and divided into segments based on sex and population ratios in stage 2. Systematic sampling was then performed to select 20 households within each segment, from which participation was requested of all individuals residing within the households who were at least one year of age. In 2010, 8,985 (81.9%) of 10,938 individuals participated in the survey, and in 2011, 8,519 (80.4%) of 10,589 individuals participated in it [2, 21]. Our final study population included 6,769 individuals aged ≥40 years.

**Measures. HRQOL and AUDIT**. Data regarding the health-related behaviors and variables were collected using two self-report questionnaires, the AUDIT and the EQ-5D, during the 2010 and 2011 KNHANES. The participants’ HRQOL was evaluated using the EQ-5D [22], a standardized instrument developed by the EuroQol Group [23] that measures five dimensions of HRQOL: mobility, self-care, usual activity, pain/disability, and anxiety/depression. Participants respond to each item by selecting the response choices, “none,” “moderate,” or “severe.” The total score is calculated using a quality weight-scoring system especially developed for the South Korean population, that ranges from 0 to 1, with a higher score indicating a better HRQOL [2,24].

Alcohol consumption patterns were classified into four zones using WHO-developed guidelines for the AUDIT score classification [25]. In accordance with the guidelines, zones I, II, III, and IV consist of total AUDIT scores of 0–7, 8–15, 16–19, and 20–40, respectively, with a higher score and zone number indicating a higher level of alcohol consumption and a greater risk of alcohol-related impairment. A score of 8 or above indicates hazardous and harmful alcohol consumption, and a score of 20 or above indicates alcohol dependency [25].

**Measures of covariates**. The health interview portion of the KNHANES was conducted by interviewers specifically trained to collect data accurately about the participants’ general characteristics and medical history. A report verifying the quality control measures that were undertaken with the persons conducting the surveys was posted on the KNHANES homepage to convey the validity of the data collected in the health interview, health behavior, and health examination surveys.

The general characteristics that were measured included age, body mass index (BMI), and marital, educational, and financial status. Age was considered a continuous variable and BMI was calculated as weight in kilograms divided by height in meters squared (kg/m^2^). The identification of marital status was limited to one of two options, as the only survey responses presented were “cohabiting” or “single.” Educational status was classified as high if the respondent had pursued education beyond middle school (above the ninth grade). Household economic status was categorized by grouping the participants by gender and age group in 5-year intervals. Each participant’s income was calculated by dividing the total household income by the square root of the number of members in the household.

The health-related behaviors that were measured included self-reports of the average number of hours of sleep, regular exercise status, smoking status, and level of alcohol consumption. Regular exercise was defined as strenuous physical activity performed for at least 20 min at a time, at least three times a week. Smoking status was determined by the participants’ self-reports of whether they had never smoked, had smoked in the past, or were current smokers. The amount of pure alcohol consumed was calculated in grams per day, according to the average number of alcoholic beverages consumed, and the frequency of alcohol consumption. Participants who consumed an average of 1–15 g/day of alcohol were considered mild-to-moderate drinkers, while those who consumed more than 30 g/day were considered heavy drinkers [26].

The prevalence of chronic disease was used as an indicator of health status. The medical history of each participant was evaluated to identify the presence of circulatory disease (diagnosis of hypertension, dyslipidemia, or stroke), musculoskeletal disease (diagnosis of osteoarthritis or rheumatoid arthritis), respiratory disease (diagnosis of pulmonary tuberculosis or asthma), liver disease (diagnosis of hepatitis B or C, or hepatocirrhosis), cancer (diagnosis of stomach, liver, colon, breast, cervix, lung, or thyroid cancer), diabetes mellitus, and depression.

### Statistical Analysis

**Fig 1:**
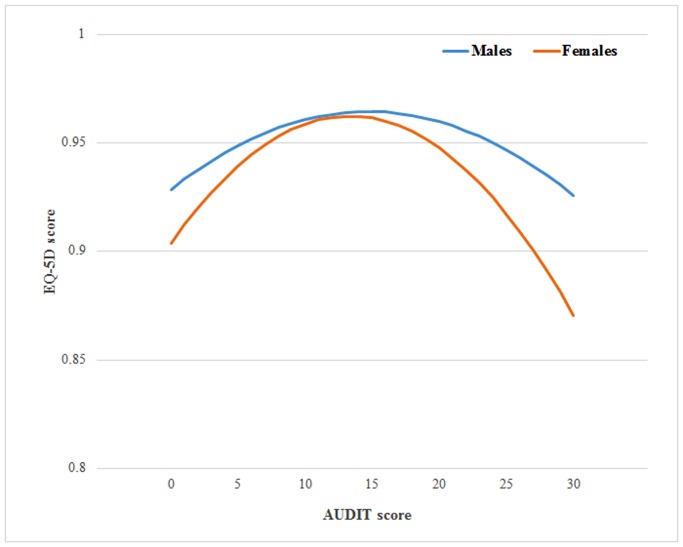
EuroQol 5-Dimension (EQ-5D) Scores by Alcohol Consumption Pattern and Sex. AUDIT: Alcohol Use Disorders Identification Test.

SAS software (ver. 9.3; SAS Institute Inc., Cary NC, USA) was used for all statistical analyses by applying the sampling weights that had been determined from the analysis of the sampling and response rates in the original data from the survey. The results of the AUDIT and EQ-5D measures of continuous variables are presented as mean (standard error [SE]) values, and the results of the EQ-5D subscale measures of categorical variables are presented as percentages (SE). A comparison of participants according to the alcohol consumption pattern and sex, in terms of general characteristics, health-related behaviors, health status, and EQ-5D score, was performed using the χ^2^ test and F-test, followed by the Scheffe test as a post-hoc test. Polynomial regression analysis was performed to evaluate the relationship between the EQ-5D scores and alcohol consumption patterns, and the predicted means are provided in Fig. 1. Multiple logistic regression analysis was conducted to evaluate the relationship between the EQ-5D subscales and alcohol consumption patterns in terms of the odds ratio (OR) and 95% confidence interval (CI). Model 1 was developed by adjusting for age and BMI; model 2 by adjusting for age, BMI, and economic, educational, regular exercise, and smoking status; and model 3 was developed by adjusting for age, BMI, and economic, educational, regular exercise, and smoking status; and the prevalence of chronic disease.

## Results

### Alcohol Consumption Patterns and EQ-5D Scores

The distribution of AUDIT and EQ-5D scores by sex are shown in Table 1. The average AUDIT score for the men was 10.0 (0.2), which was significantly higher than that for women, (3.1 [0.1]; p < 0.001). The analysis of the alcohol consumption patterns, as determined by the AUDIT scores, revealed that 44.6%, 30.2%, 13.3%, and 11.9% of men were included in zones I, II, IV, and III, respectively. In contrast, 88.3%, 9.5%, 1.2%, and 1.0% of the women met the criteria for zones I, II, IV, and III, respectively, yielding a significant intersex difference regarding the classification into zone I (p < 0.001). Likewise, a significant difference was found between the average EQ-5D score for men (0.95 [0.0]) and women (0.92 [0.0]; p < 0.001). The analysis of participants who selected “moderate” or “severe” in response to the EQ-5D items revealed sex differences in every dimension except for self-care. Statistically significant differences were found between the proportions of men and women who rated their current impairment in mobility, impairment of performance of usual activities, and experience of pain/discomfort as “moderate” or “severe” (p < 0.001).

**Table 1:** Distribution of alcohol consumption patterns and the EuroQol 5-Dimension (EQ-5D) scores by sex.

Variable		Men (n = 3,408)	Women (n = 3,361)	P-value
		Mean (SE) or % (SE)	Mean (SE) or % (SE)	
AUDIT score		10.0 (0.2) *	3.1 (0.1) *	<0.001
	Zone I (0–7)	44.6 (1.0)	88.3 (0.8)	<0.001
	Zone II (8–15)	30.2 (1.1)	9.5 (0.7)	
	Zone III (16–19)	11.9 (0.7)	1.0 (0.2)	
	Zone IV (20–40)	13.3 (0.9)	1.2 (0.3)	
EQ-5D		0.95 (0.0) *	0.92 (0.0) *	<0.001
	Mobility impairment	12.6 (0.7)	20.1 (0.9)	<0.001
	Self-care impairment	4.0 (0.4)	4.6 (0.4)	0.255
	Usual activity impairment	8.7 (0.6)	11.9 (0.7)	<0.001
	Pain/discomfort	18.8 (0.8)	29.9 (1.0)	<0.001
	Anxiety/depression	8.4 (0.6)	14.1 (0.8)	<0.001

* Note: Mean (SE)

### General Characteristics, Physical Health Status, and Health-Related Behaviors by Alcohol Consumption Pattern and Sex

Differences in general characteristics, physical health status, chronic disease, and the EQ-5D score according to the alcohol consumption patterns and sex are presented in Table 2. The men experiencing severe alcohol-related impairment were more likely to be younger, with the mean age of men in zones II, III, and IV being significantly lower than the men in zone I (p < 0.001). In contrast, the men experiencing severe alcohol-related impairment were more likely to have a higher BMI, with the mean BMI of men in zones II, III, and IV being significantly higher than that the BMI of men in zone I (p = 0.002). Regarding health status, the prevalence of musculoskeletal disease (p = 0.014), cancer (p = 0.028), and depression (p = 0.019) were significantly higher in men in zone I, (i.e., those with the lowest level of alcohol consumption), compared to the men in zones II, III, and IV. Regarding educational status, the percentage of men with a middle school diploma or lower educational level, was significantly higher in zone I and zone IV than in zone II (p < 0.001). Regarding health-related behaviors, the percentage of men in zones III and IV who were current smokers was found to be significantly higher than that of men in zones I and II (p < 0.001) The percentage of men who currently smoked and were heavy drinkers (consumed ≥ 30 g/day) increased in accordance with the extent of their alcohol-related impairment (p < 0.001). However, no significant differences were found among the zones regarding the number of hours of sleep, residential area, or marital or regular exercise status.

**Table 2:** General characteristics, physical health status, and health-related behaviors by alcohol consumption patterns and sex.

Variables		Men (n = 3,408)					Women (n = 3,361)				
		Zone I (n = 1,697)	Zone II (n = 974)	Zone III (n = 368)	Zone IV (n = 369)	P-value (Sheffe)	Zone I (n = 3,033)	Zone II (n = 264)	Zone III (n = 34)	Zone IV (n = 30)	P-value (Sheffe)
		Mean (SE) or % (SE)					Mean (SE) or % (SE)				
Age		57.0 (0.4) *	52.8 (0.4) *	51.9 (0.5) *	51.7 (0.6) *	<0.001 (II, III, and IV ˂ I)	54.6 (0.3) *	51.1 (0.6) *	46.1 (1.2) *	48.7 (1.0) *	<0.001 (II and II ˂ I)
BMI		23.7 (0.1) *	24.5 (0.1) *	24.2 (0.2) *	24.2 (0.2) *	0.002 (II, III, and IV ˃ I)	23.9 (0.1) *	24.3 (0.3) *	24.9 (0.9) *	25.1 (0.7) *	0.019 (II, III, and IV ˃ I)
Spouse (have)		93.0 (0.8)	92.0 (1.2)	93.4 (1.6)	89.7 (2.2)	0.316	79.6 (0.9)	76.8 (3.1)	92.2 (4.3)	91.1 (5.2)	0.093
Living area (rural)		25.7 (2.7)	22.8 (2.8)	26.7 (3.8)	27.8 (4.3)	0.427	23.8 (2.4)	22.0 (4.8)	18.8 (7.5)	17.6 (8.0)	0.820
Educational status (≤9 years)		39.3 (1.6)	32.4 (2.0)	31.1 (2.8)	38.2 (3.1)	0.008	50.7 (1.4)	54.8 (3.9)	51.0 (9.8)	60.4 (10.3)	0.580
Economic status (lowest)		23.1 (1.3)	14.5 (1.3)	7.7 (1.3)	16.1 (2.3)	<0.001	20.9 (1.0)	16.5 (3.4)	7.9 (4.3)	29.7 (10.1)	0.183
Sleep time		6.9 (0.0) *	6.8 (0.0) *	6.8 (0.1) *	6.8 (0.1) *	0.273	6.6 (0.0) *	6.6 (0.1) *	6.5 (0.2) *	6.8 (0.3) *	0.761
Regular exercise (yes)		21.0 (1.3)	24.4 (1.6)	22.8 (2.4)	28.4 (3.1)	0.054	18.8 (0.9)	23.1 (3.0)	47.8 (9.8)	22.1 (8.0)	0.002
Smoking status	Never smoked	26.9 (1.7)	14.6 (1.4)	13.9 (2.4)	10.4 (2.4)	<0.001	94.9 (0.5)	80.3 (3.1)	65.4 (8.8)	71.2 (9.2)	<0.001
	Ex-smoker	32.1 (2.0)	23.8 (2.0)	22.9 (3.1)	18.0 (2.7)		1.8 (0.3)	4.4 (1.7)	6.7 (6.2)	4.6 (4.5)	
	Current smoker	41.0 (1.9)	61.6 (2.2)	63.2 (3.4)	71.5 (3.2)		3.3 (0.4)	15.3 (2.7)	27.9 (10.2)	24.2(8.5)	
Alcohol	None	28.2 (1.4)	.	.	.	<0.001	24.4 (1.0)	.	.	.	<0.001
Consumption	Mild to moderate	69.6 (1.4)	49.7 (1.9)	24.4 (2.4)	8.7 (1.8)		75.3 (1.0)	69.7 (3.1)	34.4 (11.2)	34.7 (9.8)	
	Heavy	2.2 (0.4)	50.3 (1.9)	75.6 (2.4)	91.3 (1.8)		0.3 (0.1)	30.3 (3.1)	65.6 (11.2)	65.3 (9.8)	
Chronic disease	Circulatory	31.5 (1.4)	33.8 (1.8)	28.3 (2.6)	27.8 (2.9)	0.200	32.4 (1.0)	25.0 (3.1)	19.8 (7.4)	33.4 (11.3)	0.130
	Musculoskeletal	6.4 (0.7)	4.9 (0.8)	4.1 (1.0)	2.6 (0.8)	0.014	21.8 (0.9)	12.8 (2.2)	6.9 (3.7)	8.8 (4.7)	<0.001
	Respiratory	9.9 (0.9)	8.2 (1.1)	6.6 (1.4)	8.5 (1.7)	0.315	8.6 (0.6)	5.7 (1.5)	7.6 (3.9)	18.4 (9.3)	0.129
	Liver	2.5 (0.5)	1.5 (0.5)	0.8 (0.4)	3.3 (1.2)	0.081	6.3 (0.5)	7.3 (1.7)	4.7 (3.5)	17.8 (7.0)	0.058
	Cancer	4.1 (0.5)	1.6 (0.5)	2.9 (1.2)	2.0 (0.9)	0.028	5.0 (0.5)	2.3 (0.8)	.	4.0 (2.9)	.
	Diabetes	13.4 (1.0)	11.1 (1.2)	13.0 (2.2)	10.7 (1.9)	0.433	13.2 (0.7)	12.3 (2.1)	9.2 (5.0)	8.9 (7.9)	0.871
	Depression	4.4 (0.6)	2.0 (0.7)	3.9 (1.1)	1.6 (0.7)	0.019	1.7 (0.3)	2.3 (1.0)	.	1.7 (1.7)	.

* Note: Mean (SE)

Many of the findings for the female participants were similar to those of the male participants. The mean age of the women with severe alcohol-related impairment was significantly lower than that of those without impairment, with the mean age of the women in zones II and III being significantly lower than that of the women in zone I (p < 0.001). Likewise, the women experiencing severe alcohol-related impairment were more likely to have a higher BMI, with the mean BMI of women in zones II, III, and IV being significantly higher than that the mean BMI of the women in zone I (p = 0.019). Similar to the male participants, the percentage of female participants in zones III and IV, who were current smokers was significantly higher than that of the women in zones I and II (p < 0.001), and the percentage of women who were current smokers and heavy drinkers (consumed ≥ 30 g/day), increased in accordance with the extent of alcohol-related impairment (p < 0.001). Although the percentage of women performing regular exercise was the highest in zone III and lowest in zone I (p = 0.002), no significant differences were found in the number of hours of sleep. Similar to the findings for the men in this study, the women in zone I with the lowest level of alcohol consumption had a significantly higher prevalence of musculoskeletal disease than those in zones II, III, and IV (p < 0.001). However, no significant differences were found among the zones regarding residential area or marital, educational, or economic status.

### EQ-5D Scores by Alcohol Consumption Pattern and Sex

The differences identified in the EQ-5D scores according to alcohol consumption pattern and sex are shown in Table 3 and Fig. 1. The results of our analysis suggest the existence of an inverted U-shaped relationship between the total AUDIT and EQ-5D scores. Specifically, they indicate that the EQ-5D score correlates with the total AUDIT score until reaching scores of 15 and 13 for men and women, respectively, and then diverges from it. Fig. 1 shows the results of the regression analyses, which were calculated as y = -0.0001661 x^2^ + 0.004885 x + 0.9285189 for men and y = -0.0003308 x^2^ + 0.0088202 x + 0.9035745 for women, and yielded a regression coefficient that was statistically significant (p < 0.001).

**Table 3:** EQ-5D subscale score by alcohol consumption patterns and sex.

Subscale	Male (n = 3,408)					Female (n = 3,361)				
	Zone I (n = 1,697)	Zone II (n = 974)	Zone III (n = 368)	Zone IV (n = 369)	P-value	Zone I (n = 3,033)	Zone II (n = 264)	Zone III (n = 34)	Zone IV (n = 30)	P-value
	Mean (SE) or % (SE)					Mean (SE) or % (SE)				
	0.94 (0.0) *	0.96 (0.0) *	0.97 (0.0) *	0.95 (0.0) *	0.004 (I < III)	0.92 (0.0) *	0.94 (0.0) *	0.96 (0.0) *	0.96 (0.0) *	<0.001 (I ˂ II, III, IV)
Mobility impairment	15.2 (1.0)	10.1 (1.1)	8.3 (1.6)	13.6 (2.2)	0.002	21.2 (1.0)	13.0 (2.2)	6.7 (3.6)	6.3 (3.7)	<0.001
Self-care impairment	5.6 (0.7)	3.2 (0.7)	0.6 (0.4)	3.5 (1.1)	<0.001	4.9 (0.4)	2.8 (1.0)	1.2 (1.2)	23.1 (0.7)	<0.001
Usual activity impairment	11.1 (0.9)	6.9 (1.0)	4.8 (1.4)	8.4 (1.8)	0.001	12.5 (0.7)	9.0 (1.7)	2.9 (2.1)	1.7 (1.7)	<0.001
Pain/discomfort	20.6 (1.2)	17.4 (1.5)	13.5 (2.0)	20.3 (2.5)	0.040	31.1 (1.1)	23.3 (3.0)	14.5 (5.9)	8.4 (4.6)	<0.001
Anxiety/depression	8.4 (0.9)	6.5 (1.0)	5.2 (1.5)	15.7 (2.4)	<0.001	13.7 (0.8)	17.8 (3.0)	10.7 (5.1)	15.0 (6.8)	0.398

* Note: Mean (SE)

A number of differences were observed regarding the EQ-5D subscales. The HRQOL for men in zone III was significantly higher than that of men in zone I (p = 0.004), and the percentage of men experiencing mobility impairment was significantly higher in zones I and IV than in zones II and III (p = 0.002). The percentage experiencing self-care impairment was significantly higher in zone I compared to zones II, III, and IV (p < 0.001); and, the percentages experiencing impairment in performing usual activities and experiencing pain/discomfort were significantly higher in zones I and IV compared to zones II and III (p = 0.001 and p = 0.040, respectively). In contrast, the percentage experiencing anxiety/depression was significantly higher in zone IV than in zones I, II, and III (p < 0.001).

The findings for the female participants were somewhat consistent with those of the males. The HRQOL of the women in zones II, III, and IV were significantly higher than that of the women in zone I (p < 0.001). With regard to disability, the proportions of women experiencing mobility impairment, self-care impairment, impairment in performing usual activities, and pain/discomfort were significantly higher in zone I than in zones II, III, and IV (p = 0.001, p < 0.001, p = 0.006, and p = 0.001, respectively). However, no significant differences were found among the zones regarding the rates of anxiety/depression.

### Association between Alcohol Consumption Patterns and the EQ-5D Subscales by Sex

Table 4 shows the relationship between alcohol consumption patterns and the EQ-5D subscales by sex. Although intersex differences were identified, they did not reach a level of statistical significance. For both sexes, an analysis of the proportions of subjects answering either “moderate” or “severe” to the EQ-5D items for alcohol consumption patterns indicated the existence of an inverted U-shaped relationship among zones I, II, and IV, with zone III at the center. For men, the mobility, pain/discomfort, and anxiety/depression subscales slightly varied among models 1, 2, and 3. However, the OR for anxiety/depression among men in zone IV was 3.52 times higher (95% CI, 1.59–7.81) compared to the men in zone III (reference point) (p < 0.001). The OR for self-care impairment in model 1 was 5.69 (95% CI, 1.04–30.90) for men in zone I and 7.85 (95% CI, 1.37–44.78) for men in zone IV (p < 0.001). The OR for pain/discomfort in model 2 was 2.56 (95% CI, 1.01–6.44) for women in zone I compared to those in zone III (p < 0.001).

**Table 4:** Associations between alcohol consumption pattern and EQ-5D subscales by sex.

Subscale		Male (n = 3,408)				Female (n = 3,361)			
		Zone I (n = 1,697)	Zone II (n = 974)	Zone III (n = 368)	Zone IV (n = 369)	Zone I (n = 3,033)	Zone II (n = 264)	Zone III (n = 34)	Zone IV (n = 30)
		Adjusted OR (95% CI)				Adjusted OR (95% CI)			
Mobility impairment	Model 1	1.08 (0.59–1.95)	1.06 (0.60–1.85)	1	**2.36 (1.20–4.65)** *	1.54 (0.48–4.93)	1.17 (0.35–3.88)	1	0.57 (0.08–3.74)
	Model 2	1.12 (0.62–2.01)	0.97 (0.55–1.73)	1	**2.10 (1.05–4.20)** *	2.27 (0.62–8.25)	1.48 (0.40–5.50)	1	0.54 (0.07–3.82)
	Model 3	1.10 (0.60–2.01)	0.94 (0.52–1.69)	1	**2.18 (1.09–4.37)** *	1.82 (0.56–5.92)	1.31 (0.39–4.40)	1	0.44 (0.06–3.03)
Self-care impairment	Model 1	**5.69 (1.04–30.90)** *	5.23 (0.97–28.03)	1	**7.85 (1.37–44.78)** *	1.05 (0.13–8.15)	1.11 (0.12–9.53)	1	.
	Model 2	5.20 (0.97–27.86)	4.92 (0.90–26.80)	1	**6.76 (1.17–38.88)** *	1.41(0.16–12.01)	1.29 (0.14–11.7)	1	.
	Model 3	5.06 (0.92–27.84)	4.62 (0.82–25.96)	1	**6.88 (1.20–39.55)** *	1.07 (0.12–8.97)	0.93 (0.10–8.64)	1	.
Usual activity	Model 1	1.53 (0.73–3.22)	1.33 (0.62–2.81)	1	2.09 (0.89–4.88)	1.74 (0.38–8.04)	1.76 (0.36–8.44)	1	.
impairment	Model 2	1.48 (0.69–3.14)	1.26 (0.57–2.79)	1	1.78 (0.75–4.23)	2.56 (0.52–12.57)	2.21 (0.44–11.13)	1	.
	Model 3	1.40 (0.65–3.02)	1.18 (0.52–2.71)	1	1.77 (0.75–4.17)	2.13 (0.46–9.80)	1.92 (0.40–9.10)	1	.
Pain/discomfort	Model 1	1.25 (0.80–1.95)	1.27 (0.80–2.01)	1	**1.88 (1.12–3.17)** *	1.85 (0.73–4.70)	1.45 (0.55–3.83)	1	0.38 (0.07–1.98)
	Model 2	1.25 (0.79–1.97)	1.27 (0.80–2.01)	1	**1.80 (1.07–3.05)** *	**2.56 (1.01–6.44)** *	1.76 (0.66–4.65)	1	0.36 (0.06–2.02)
	Model 3	1.22 (0.78–1.93)	1.25 (0.79–1.98)	1	**1.84 (1.09–3.10)** *	2.34 (0.95–5.73)	1.70 (0.65–4.41)	1	0.32 (0.05–1.76)
Anxiety/depression	Model 1	1.10 (0.52–2.31)	1.24 (0.57–2.70)	1	**3.52 (1.59–7.81)** *	1.09 (0.38–3.12)	1.49 (0.49–4.54)	1	1.21 (0.25–5.76)
	Model 2	1.06 (0.50–2.24)	1.17 (0.53–2.56)	1	**3.08 (1.38–6.90)** *	1.15 (0.39–3.39)	1.53 (0.49–4.74)	1	1.05 (0.20–5.44)
	Model 3	1.01 (0.48–2.13)	1.15 (0.52–2.50)	1	**3.16 (1.43–6.94)** *	1.04 (0.37–2.92)	1.41 (0.47–4.25)	1	0.81 (0.18–3.54)

Model 1: adjusted for age and BMI

Model 2: adjusted for age, BMI, and economic, educational, regular exercise, and smoking status

Model 3: adjusted for age, BMI, economic, educational, regular exercise, and smoking status, and the prevalence of chronic disease

*p < 0.001

## Discussion

In 2011, the WHO (2011) adopted the “Global strategy to reduce the harmful use of alcohol” and required all member states to draft and implement a national strategy to reduce the harmful consumption of alcohol. Despite the growing international interest in this issue, South Korea continues to experience a steady increase in alcohol consumption, which most likely is because of the population’s low level of awareness of alcohol’s harmful effects and South Korea’s traditionally high level of alcohol consumption [3,6]. The current study yielded several findings that may assist in addressing this problem.

The total AUDIT score was higher in men than in women, excluding zone I. In a previous study of a general population-based Norwegian sample [5], the total AUDIT score of the men was 6.54 (4.30), and it was higher than that of the women, which was 4.24 (3.29). Although the Norwegian study also found a higher AUDIT score in men, the gender difference was more pronounced in the present study. Heavy alcohol consumption is widespread throughout South Korea and it often is expected in social and professional settings for men [27], while it is less socially acceptable for women. In addition, this study found that women, compared to men, had lower HRQOL and that the percentages of “moderate impairment” and “severe impairment” responses of women on all the subscales were high. These results contradict those of a previous study, which found differences in HRQOL using the same instrument but not intersex differences [5]. The findings are in agreement with studies conducted in Spain [19] and China [28] using the same instrument. The observed differences between the men and women in the present study might be attributable to the fact that men appear to have a less serious attitude about physical disease and illness and show fewer displays of emotion (e.g., crying) in reaction to loss; they also are less likely to seek treatment for illness and disease.

As explained previously, it is difficult to compare the findings regarding general characteristics, health-related behaviors, and chronic diseases by sex and alcohol consumption patterns between different studies, due their different categorizations of alcohol consumption patterns. In this study, differences in age, BMI, educational status, and economic status were found among the men, while only differences in age and BMI were found among the women. Consistent with a previous study [17] that reported an inverse relationship between increases in harmful alcohol consumption and age, the average age of both men and women was found to be lower in the zones associated with severe alcohol-related impairment. However, the differences found among the zones regarding other socio-demographic factors differed from those identified in previous studies [17,29]. To explain the differences identified in this study, it is necessary to examine the reasons for excessive alcohol consumption and/or the lack of alcohol consumption in the lowest age quartile within a cultural context.

Consistent with previous studies [8,9,17] that found a relationship between harmful consumption of alcohol and the exacerbation of negative health-related behaviors, the percentages of current smokers and heavy drinkers in the current study were found to be highest in zone IV for both men and women. However, the percentage of women engaging in regular exercise was found to be highest in zone III, a finding that was different from what was anticipated. Given the results of previous studies [8,9], it was expected that the participants with positive health-related behavior would have positive drinking patterns, such that the percentage of women engaging in regular exercise would be the highest in zone I. However, for both men and women, the mean BMI in zone I was found to be the lowest, which was consistent with the results of a previous study [30], which reported that higher alcohol intake was associated with greater total and central adiposity. In terms of chronic disease, the rates of all chronic diseases, excluding liver-related diseases associated with alcohol consumption, were found to be the highest in zone I and the lowest in zone IV, which was an unexpected finding. However, this finding may be explained by the fact that the prevalence of chronic diseases, including hypertension, osteoarthritis, diabetes, and cancer, generally increase exponentially with age [2,21], and that the participants in zone I were significantly older than those in zone IV. Thus, this finding might be attributable to the results of the univariate analysis without adjusting the data for age and occupational status.

In support of this hypothesis, the analysis of the non-age-adjusted data revealed the existence of an inverted U-shaped relationship between the total AUDIT score by sex and the EQ-5D score, consistent with previous studies [5,31]. Moreover, the inflection points of the AUDIT score at which the HRQOL subscale began to change were found to be 15 for men and 13 for women, both of which are higher than the cutoff value of 8, as stipulated in the AUDIT guidelines [25]. This finding is partially consistent with that of a previous study using the WHO Quality of Life instrument (WHOQOL-BREF). This tool is a cross-cultural assessment of quality of life that examines the domains of physical health, psychological health, social relationships, and environmental factors [32]. The study found that participants in zone II had the highest score for physical health [5]. Although it is difficult to generalize from these findings, the tendency of middle-aged men to drink alcohol as a means of stress relief and the tendency of non-abusers of alcohol to focus on maintaining a high quality of life [4] may indicate the need to use a higher cutoff value for this population. The examination of this possibility would require further research on the relationship between alcohol consumption and sex and age. The final notable finding was that the analysis of data adjusted for age and other variables revealed that the HRQOL of men was likely to increase between zones I and III, but to decrease subsequently between zones III and IV, to the extent that the difference between zones III and IV became statistically significant. On the other hand, the percentage of women experiencing pain/discomfort was found to be significantly lower in zone I compared to those in zones II, III, and IV (p < 0.001).

Although several findings of this study were not statistically significant, overall, they support the findings of previous studies, including an association between high levels of alcohol consumption and poor HRQOL [5,29,33], and self-reports of better physical HRQOL by individuals who consume alcohol, including moderate and heavy drinkers, compared to individuals who consume no alcohol [19]. Nevertheless, no clear alcohol consumption patterns could be identified among the women in this study, which might have been due to the very limited number of females in zones III and IV. Moreover, our results also revealed slight differences in the ORs for the risk of experiencing alcohol-related impairment among the three models, with the OR values in model 1, which was adjusted for only age and BMI, found to be the highest, and the OR values in model 3, which was adjusted for all variables, found to be the lowest. The differences in the ORs of the three models are considered to result from the influence of the covariates on HRQOL. In addition, different covariates that affected HRQOL according to gender were identified.

Among the alcohol-related impairments among the men [25] in zone IV, the most frequently reported impairment was experiencing pain/discomfort (20.3%), followed by anxiety/depression (15.7%), impairment in mobility (13.6%), impairment in performing usual activities (8.4%), and impairment in providing self-care (3.5%) [7]. Similarly, a previous study targeting patients diagnosed with alcohol dependency, in which 72.8% of the participants were male, found that the most commonly reported impairment was experiencing anxiety/depression (49.0%), followed by pain/discomfort (42.6%), impairment in usual activities (28.7%), impairment in mobility (15.5%), and impairment in providing self-care (11.7%). The impairment in self-care was the least frequently reported impairment [25]. One explanation why the proportion of men who reported experiencing self-care impairment was much higher in the previous study than in the present study may be that the participants in the previous study were hospitalized patients previously diagnosed with alcohol dependency, while those in the present study were individuals living in the community. Moreover, a likely explanation for why the ORs for experiencing impairment in self-care among the men was higher compared to the ORs for other subscales, is that the number of participants in zone IV who reported impairment in self-care was much lower than the number of participants who reported impairment in other areas. These findings indicate that the perception of adequate self-care among alcohol-dependent individuals might shift over the years, due to their adaptation to their alcohol-impaired condition [7]. Of course, these differences may be due to differences with respect to the severity of the diseases in the two populations. Therefore, caution should be used in generalizing the results of the present study.

Alternatively, these findings may reflect measurement bias. As the EQ-5D is a self-report questionnaire, and as patients may be reluctant to report experiencing impairments in basic life activities, they may not answer all of items completely and honestly. However, even a conservative interpretation of the study’s findings supports the hypothesis that the HRQOL of individuals who consume moderate amounts of alcohol is higher than the HRQOL of those who consume no alcohol (0 g/day) or large amounts of alcohol (≥ 30 g/day). Although participants in zone I were found to have a relatively poor HRQOL, this finding is limited by the fact that this study did not distinguish between nondrinkers who refrained from alcohol consumption due to health and/or other external conditions and those who refrained for other reasons. The high prevalence of chronic diseases, except for liver-related diseases, among the participants in zone I, who were significantly older than those in the other zones, could be explained by the natural increase in chronic disease prevalence with age. It also could be explained, in part, by the inclusion of participants who refrain from alcohol consumption permanently or temporarily due to the presence of chronic disease, which consequently, would decrease their HRQOL. Therefore, longitudinal research examining different variables associated with alcohol consumption patterns should be conducted in the future to determine the precise correlations between alcohol consumption patterns and HRQOL, and to identify whether any causal relationships exist. Such research also should examine alcohol consumption patterns, especially those of moderate drinkers, in terms of various aspects of the alcohol consumption culture, including but not limited to preferred alcohol types; alcohol consumption frequency; and differences in patterns among each level of drinker by sex, nationality, and individual characteristics.

There are a number of limitations associated with this study. First, the analysis of secondary data, namely the KNHANES raw data, which were collected for purposes other than those of this study, is a major limitation. Using secondary rather than primary data poses intrinsic limitations, such as the lack of inclusion of all of the relevant variables and the use of different operational definitions of the relevant variables in the original study. Second, the use of a cross-sectional design is another limitation, as this makes it difficult to determine the causal relationship between alcohol consumption patterns and HRQOL. Thus, we recommend that longitudinal studies examining the temporal relationship between the two variables should be conducted in the future. In the case of the women’s alcohol consumption patterns, the results should be interpreted with caution because the percentages of women in zones III and IV were very low, and they exhibited additional differences (in education, exercise, and income) from the rest of the study population who drank less, thereby limiting the generalizability of the study’s results. Despite these limitations, a major strength of this study is that it is the first to examine the relationship between alcohol consumption patterns and HRQOL in Korean men and women using a representative nationwide sample of Korean adults.

## Conclusions

The findings of this study indicate that the HRQOL of moderate alcohol drinkers is higher than that the HRQOL of non-drinkers and heavy drinkers. It is believed that these findings will be valuable in designing appropriate interventions to improve HRQOL that has been impaired by the harmful use of alcohol. It also should be useful for comparisons of HRQOL among different countries, and for the implementation of alcohol-related health projects. The application of these findings should be verified by future longitudinal studies examining the existence of a causal relationship between alcohol consumption patterns and HRQOL, while considering the culture of alcohol consumption and differences in consumption by sex, nationality, and individual characteristics.
